# Beyond Separate Emergence: A Systems View of Team Learning Climate

**DOI:** 10.3389/fpsyg.2019.01441

**Published:** 2019-07-03

**Authors:** Jean-François Harvey, Pierre-Marc Leblanc, Matthew A. Cronin

**Affiliations:** ^1^HEC Montréal, Montreal, QC, Canada; ^2^Université de Montréal, Montreal, QC, Canada; ^3^George Mason University, Fairfax, VA, United States

**Keywords:** team learning, systems view, team emergent states, team leadership, team dynamics, team monitoring

## Abstract

In this paper, we consider how the four key team emergent states for team learning identified by Bell et al. (2012), namely psychological safety, goal orientation, cohesion, and efficacy, operate as a system that produces the team’s learning climate (TLC). Using the language of systems dynamics, we conceptualize TLC as a stock that rises and falls as a joint function of the psychological safety, goal orientation, cohesion, and efficacy that exists in the team. The systems approach highlights aspects of TLC management that are traditionally overlooked, such as the simultaneous influence of and feedback between the four team emergent states and the inertia that TLC can have as a result. The management of TLC becomes an issue of controlling the system rather than each state as an independent force, especially because changing one part of the system will also affect other parts in sometimes unintended and undesirable ways. Thus the value is to offer a systems view on the leadership function of team monitoring with regards to team emergent states, which we term *team state monitoring*. This view offers promising avenues for future research as well as practical wisdom. It can help leaders remember that TLC represents an equilibrium that needs balance, in addition to pointing to the various ways in which they can influence such equilibrium.

## Introduction

Team emergent states are defined in terms of beliefs that team members hold about the team’s goals, team member abilities, and interpersonal norms. They emerge early after team formation and continue to develop over time as the team’s work unfolds (Marks et al., 2001; Cronin et al., 2011; Edmondson and Harvey, 2018). They tend to stabilize as beliefs become relatively coherent across team members (Kozlowski and Chao, 2012), ultimately guiding behaviors within the team (e.g., Edmondson, 1999). Yet their emergence is described as dynamic because they form in response to experiences and observations of team member interactions, and these experiences and observations both shape and are shaped by the accumulating beliefs. We know a fair amount about what makes particular team states emerge, and how team leadership can influence such emergence (e.g., Edmondson and Harvey, 2017), but we know significantly less about the feedback among team states when they are linked as a system, and what this means for team leadership seeking to control that system.

In this paper, we focus on team learning because it is one of the most critical team processes, and team leaders have significant impact on creating conditions that support it (Koeslag-Kreunen et al., 2018). We draw from Bell et al. (2012) to consider four key emergent states for team learning, namely psychological safety, goal orientation, cohesion, and efficacy, and we argue that collectively these states bring about the team’s learning climate (TLC). We conceptualize TLC as a capacity that rises and falls as a joint function of the psychological safety, goal orientation, cohesion, and efficacy that exist in the team. If the four emergent states can increase or decrease the level of TLC, then collectively TLC can be conceptualized as a control system (cf. Vancouver, 2005). If the level in any one component of the system (e.g., cohesion) affects but is also affected by other components (e.g., psychological safety), then there is feedback in this system. If the levels of a component can persist over time, then there is inertia. It is these two conditions that make a system dynamic (Cronin and Vancouver, 2018), and dynamics increase the challenge of maintaining control of a system (Cronin et al., 2009).

Because leadership activities may influence multiple team emergent states at once, it is fundamental to take a systems view (Sterman, 2000) of how the various states affect the rates of increase and decrease to TLC. It is when leaders are conscious of their influence on emergent states as a system that they come to realize that their interventions can simultaneously affect the various parts of the system in distinct ways (Shuffler et al., 2018), or that their interventions can have little impact because of the inertia found in the system (Ericksen and Dyer, 2004). A focus on one particular emergent state to the exclusion of others is often why practices intended to help wind up being net negative (Sterman, 2000). Leaders can overlook the side effects that would be visible had they taken a broader view of the entire system. This is particularly important in teams because most teams encounter turbulences, and it is during turbulences that their leaders intervene. It is also during such times that a leader’s focus can narrow (Staw et al., 1981).

The systems view helps further elaborate on the leadership function of team monitoring. Functional leadership doesn’t prescribe individual traits to good or bad leaders, but rather informs on the interventions required to satisfy team needs. The core idea is not to emphasize “what leaders should do” but rather “what needs to be done for effective performance” (Hackman and Walton, 1986, p. 77). From this perspective, team leadership is about identifying and solving problems with the aim of ensuring team effectiveness. Team monitoring is a key leadership function that refers to examining a team’s internal activity, progress toward the achievement of the team task, and its environment (Morgeson et al., 2010). While some studies have examined the relationship between team monitoring and team learning—e.g., both De Jong and Elfring (2010) and Otte et al. (2017) have shown the positive and significant relation between team monitoring and team reflexivity (reflexivity is a learning process)—the emergent states described as part of TLC have not been considered in any of these studies. Our systems approach helps provide an understanding of how team states influence TLC, and how TLC can be effectively controlled over time. Thus monitoring TLC is better understood when we view teams as systems where inertia and feedback inform leadership.

Specifically, we propose that *team state monitoring* is a key leadership function that encompasses the routine evaluation of how a team evolves to identify and correct dysfunctional imbalances in a collection of team states. Because we take a systems view of team emergent states’ development, we not only focus on how changes to one state might propagate throughout the system, we also consider the unintended consequences that can be created as leaders attempt to manage these states. We argue that monitoring is effectively the means to manage TLC over time, but such monitoring can be myopic and lead to actions that enhance one part of the system while degrading others. It can be beneficial, however, when leaders take a systems view.

In the sections that follow, we review the literature on team emergent states and team learning to develop a systems view of TLC. Then, we operationalize this view through a vignette that helps illustrate why it matters for team leadership before deepening the notion of team monitoring as a leadership function. This takes us to a discussion of team state monitoring and its implications for team research and leadership practice.

## Team Learning Climate

Since the seminal work of Senge (1990), learning has become a central part of the literature in management (Huber, 1991; Edmondson, 2002; Wilson et al., 2007). Many researchers and practitioners have adopted Senge’s view that organizations need to learn in order to achieve and maintain superior performance. His argument is that fixed commitment to a leader’s vision is ultimately a bad strategy. The business environment inevitably changes over time, and thus organizations need to be able to adapt. As a result, Senge advocates for developing reflection and inquiry skills throughout the organization, hence facilitating the continuous emergence of new ways of thinking. Organizations are then better able to adapt quickly and effectively by matching (or creating) radical changes in their environment. He argues that work teams are a key unit for such learning to occur in organizations because learning begins with dialogue, a dialogue that allows individuals to make sense of complex situations and discover insights not attainable individually. Team learning has since been studied extensively in organizational behavior to explain team effectiveness.

Edmondson et al. (2007) find three perspectives on team learning in the literature, and each one of them considers features of TLC to be important. The first one, outcome improvement, examines the progression that teams go through as they gain cumulative experience performing the same set of tasks. The outcome improvement research shows clearly that teams learn at a different rate, and such differences have been attributed to various factors, such as team composition stability (Edmondson et al., 2003) or communication networks (Argote et al., 2018), and the learning climate (Edmondson et al., 2001). These studies demonstrate that team performance increases over time as teams learn how to improve their coordination (Reagans et al., 2005).

The second perspective, task mastery, suggests that team learning occurs when teams develop shared knowledge about each other and the task during the process of discussing and coordinating effort. Teams are seen as information-processing systems that may be better or worse at encoding, storing, retrieving, and communicating knowledge (Hinsz et al., 1997; Wilson et al., 2007). Better teams are said to develop a more elaborate “transactive memory system,” which enhances performance on interdependent tasks (Liang et al., 1995). For instance, Ellis and colleagues define team learning as “the team’s collective level of knowledge and skill produced by the shared experience of the team members” (Ellis et al., 2003, p. 822). Interventions that involve training team members together on the task (e.g., Moreland et al., 1996) and facilitating face-to-face communication (Lewis, 2004) are demonstrative of this perspective on team learning. Scholars also find that the learning climate is a factor to consider in teams developing such shared knowledge (Hammedi et al., 2013).

Finally, the third perspective defines team learning in terms of the activities of the learning process instead of its outcomes. It is deeply rooted in the input-process-output (IPO) model first developed by McGrath (1964). In this model, team member behaviors and interactions are the processes that transform input conditions into performance outputs (e.g., Hackman and Morris, 1975). As such, team learning comprises many different sorts of learning behaviors that reflect the particular needs and goals of the specific team (Edmondson, 2002). They include four behaviors: (a) building prototypes, drawing sketches, and running trials (e.g., Lee et al., 2004), (b) questioning goals or methods to reach them, suggesting alternatives, reflecting on new information (e.g., West, 1996), (c) engaging with experienced others outside the team (e.g., Bresman, 2010), and (d) seeking information about the environment (e.g., Ancona and Caldwell, 1992). Put together, these behaviors take place inside or outside the team, and may serve exploration or exploitation purposes (see Harvey et al., 2018). In this paper, we focus on learning behaviors that take place inside the team because they are more dependent on TLC (e.g., Wong, 2004).

Drawing on these three perspectives, we define team learning as team members’ behaviors related to processing knowledge that allows the team to improve. We argue that while team leaders can control inputs, they actually spend most of their time managing processes as they change in response to alterations in tasks and environment. In other words, individuals are the agents of learning, and the agents that initiate team learning. Because of that, leaders do not really affect the individuals as much as they set up conditions that enable individual/team learning. This is why Senge (1990) suggests that leaders in organizations should first and foremost enable individuals to adopt learning behaviors within their respective teams. Such enabling conditions usually relate to the beliefs that are shared by team members with regards to the team and its task, which have been termed “team emergent states.”

### Key Team Emergent States in Support of Team Learning

A lot of the scholarly conversation on team learning focuses on understanding the conditions that facilitate learning in teams; that is, the states that emerge over time as individuals engage in teamwork and facilitate or constrain learning behaviors. The distinction between states and processes was a critical step toward understanding the dynamics of teamwork. As Marks et al. (2001) have argued, the conditions of states are what influence a team and can persist over time. For example, the level of trust today will maintain itself over time until some other process changes that level. States allow explicit consideration of inertia in contrast to processes, like communication, that only affect the team when they are engaged and thus do not have inertia. The levels in the states alter the processes that take place in the team. Continuing with our example, a high level of trust may lead to more frequent and open communication, while a low level would make communication less frequent and more guarded. Processes also change states, so the open communication may further increase the level of trust. Taken together, Marks et al. (2001) highlight the feedback between states and processes that affect the dynamics of team conditions over time.

While Marks and colleagues’ model has offered a conceptual path toward further precision in the exploration of team dynamics, much of the research that has followed does not take advantage of these. Most research focuses on the substance of emergent states, and largely studies them as moderators of other relationships without considering how they emerge and evolve in the first place (Waller et al., 2016). In particular, the ways in which emergent states dynamically interact with each other to explain certain team outcomes remains underexplored (Cronin et al., 2011), despite research demonstrating their joint effects in creating pathways that spur team learning (Harvey et al., in press). Before we can describe such dynamic interrelationships, we must briefly review the functionality of the four emergent states that have received most attention in team learning scholarship—psychological safety, goal orientation, efficacy, and cohesion (Bell et al., 2012), summarized in Table 1. It is the fact that each emergent state has a different functionality but that these states may jointly affect common processes that justifies the need to consider them as a dynamic system.

**Table 1:** Team emergent states, influences on team learning, and supportive leadership practices.

Team emergent state	Psychological safety	Goal orientation	Cohesion	Efficacy
Definition	The shared belief that the team is safe for interpersonal risk taking.	The shared belief of the extent to which a team emphasizes learning or performance goals.	The shared belief of commitment from team members to the task or to each other.	The shared belief that the team can successfully perform the task.
Influences on team learning	Moderate to high levels of psychological safety influence positively the adoption of learning behaviors.	Moderate to high levels of learning orientation influence positively the adoption of learning behaviors. High levels of learning orientation can be ineffective because teams mistakenly abandon effective strategies to pursue novel ones. Moderate to high levels of performance orientation negatively influence the adoption of team learning behaviors.	Moderate to high levels of cohesion influence positively the adoption of learning behaviors. High levels of cohesion can negatively influence the adoption of learning behaviors because teams suffer from groupthink.	Moderate to high levels of efficacy influence positively the adoption of learning behaviors. High levels of efficacy can negatively influence the adoption of learning behaviors because teams succumb to overconfidence and complacency.
Supportive leadership practices	Displaying genuine interest in team member’s particular needs and challenges in completing the task. Inviting and showing appreciation for others’ contributions. Creating clear structures. Establishing shared rewards.	Offering feedback on behaviors or reward certain outcomes. Encouraging discussion of opposing views.	Explicating shared values and articulating the team goal. Shaping leader-member relationships in ways that lower perceptions of differentiation. Requesting task-relevant information, pointing to flaws in task procedures, and questioning the team’s output.	Displaying the belief that one is capable of achieving good performance. Designing the team’s work in order to achieve early wins.

#### Psychological Safety

Edmondson (1999) has examined team psychological safety – the shared belief that a team is a safe place to take interpersonal risks – as a variable that would affect team learning. She has shown that learning behaviors translate effective team leadership into performance outcomes when team members feel able to question assumptions and discuss difficult issues. For instance, engaging in trial-and-error experimentation is extremely difficult when there is a sense that team members’ participation is being scrutinized or evaluated because chance of success is uncertain and failure is a strong possibility (Lee et al., 2004). The open discussion of errors, just as voicing ideas and concerns, requires a psychologically safe environment that encourages team members to engage in candid conversation focused on improving team task performance (Carmeli and Gittell, 2009), instead of succumbing to defensive routines such as self-censoring (Argyris, 1990).

Today, psychological safety is the most common emergent state studied in relation to team learning (Sanner and Bunderson, 2015). It has been shown to have a positive relationship with team learning in a great variety of settings (for reviews, see Edmondson and Lei, 2014; Newman et al., 2017). Companies as influential as Google have pointed to psychological safety as the most important feature of high-performing work teams (Duhigg, 2016).

Leaders can nurture psychological safety by inviting and showing appreciation for others’ contributions (Nembhard and Edmondson, 2006), creating clear structures (Bresman and Zellmer-Bruhn, 2013), and establishing shared rewards (Chen and Tjosvold, 2012). Edmondson and Harvey’s (2017) multiple case study of extreme teaming projects also offers an in-depth account of what leaders can do to foster rapport that gives rise to psychological safety. The authors find that successful project leaders are not solely focused on task completion and project progress when they interact with team members, but also display genuine interest in team members’ needs and challenges in completing the task.

Psychological safety should be thought of as having inertia. It is a belief that builds over time (Edmondson, 1999), and while behaviors can subtract from its level, the prior level should persist over time. For example, one angry outburst at a team member for a mistake would not destroy all psychological safety, though it would probably reduce the level (Edmondson, 2018). Also, a team that was temporarily disbanded and then re-assembled would be unlikely to restart from zero in terms of expectations about psychological safety.

#### Goal Orientation

Drawing on the work of Dweck (1986) and others (e.g., Button et al., 1996; VandeWalle, 1997) on individuals’ psychological traits, Bunderson and Sutcliffe (2002, 2003) have shown that teams may approach achievement situations from two angles: learning and performance. When teams are oriented toward learning, their members take a proactive approach to solving new, complex problems and are more likely to engage in behaviors that facilitate learning (Alexander and Van Knippenberg, 2014). Since they are not particularly interested in relying on prior capabilities, these teams invest considerable time and energy in planning their work (Mehta et al., 2009) and their members continue to exchange information with each other during execution (Gong et al., 2013). In contrast, in achievement situations where teams are oriented toward performance, novel or puzzling insights tend to prompt irritation or discomfort rather than enthusiasm, because they undermine the team’s strongly rooted commitment to the collective expression of competence and the favorable judgment that comes with it (Mehta and Mehta, 2018). Mistakes are far less welcome on such teams, since they prize concrete progress or tangible results. For instance, highly performance-oriented teams are unlikely to continue pursuing radical innovation after they encounter challenges, because they realize that doing so increases their chances of failure (Alexander and Van Knippenberg, 2014).

Leadership influences the emergence of a learning or performance orientation on teams. Dragoni and Kuenzi (2012) show that the leader’s individual goal orientation influences that of the team. Leaders are likely to induce learning or performance orientation when they offer feedback on behaviors or reward certain outcomes (Alexander and Van Knippenberg, 2014). For their part, Chen et al. (2011) show that leaders facilitate the emergence of a learning orientation by encouraging discussion of opposing views, while Bunderson and Boumgarden (2010) show that conflicts and disagreements between team members reduce the odds that a learning orientation will emerge within the team.

While some team research treats goal orientation as a team composition variable (an input in the ISPO model) (e.g., LePine, 2005; Porter, 2005), the research above along with several other team studies (e.g., DeShon et al., 2004; Mehta et al., 2009) conceptualize it as a team emergent state. The reason to conceptualize goal orientation as a state is that while individuals may have goal orientations when they join a team, such individual orientations are not immediately manifest by the collective, and the collective level may change over time given leadership behaviors and incentives (Dragoni and Kuenzi, 2012). Again, because team goal orientation is an intangible property, individuals’ beliefs about it are more likely to have inertia. We could also imagine team factions that diverge in their goal orientations; it would make goal orientation more “compilational” in structure (Klein and Kozlowski, 2000), but it would still make it a state with inertia.

#### Cohesion

Team cohesion, defined as the shared belief or commitment from team members to the task, or to each other, has been extensively studied (Beal et al., 2003). Both the integration or “bonding” of individual team members into the group (social cohesion) as well as their desire to accomplish the team task (task cohesion) have been argued to increase team members’ willingness to invest time and energy within the team (Hackman, 1990). This is important for team learning because adopting learning behaviors is demanding for team members (Edmondson, 2003; Edmondson and Harvey, 2018).

Leaders can play a significant role in influencing the degree of cohesion in teams. Edmondson and Harvey (2017) find that leaders may facilitate its development by explicating shared values in articulating the team goal. Similarly, Chiniara and Bentein (2018) show that shaping leader-member relationships in ways that lower perceptions of differentiation positively influences team cohesion. The degree of participation from team members in key facets of the team endeavor is another factor that affect team cohesion (Bergman et al., 2012) and that leaders can enable. Leaders can also strategically request task-relevant information, point out flaws in task procedures, and question the team’s output. Monitoring task complexity in such a way brings team members together (Kane et al., 2002).

Once again, because cohesion takes time to build (Mathieu et al., 2015) and is stored in individuals’ beliefs, we posit that it will not necessarily dissipate without some event and that it has inertia. However, such a state may have the possibility for more drastic change in a moment than, for example, goal orientation (which is rooted in individual proclivities). For example, some huge violation or betrayal by team members could destroy team cohesion (Mach et al., 2010). Yet the level of cohesion would move from its prior level to the new level, meaning that cohesion at time t+1 is a function of the event plus cohesion’s level at time t. This is how one operationalizes inertia (Cronin and Vancouver, 2018).

#### Efficacy

Researchers have theorized that team members’ confidence in their capability *vis-á-vis* one particular task—team efficacy—is an important determinant of team performance (e.g., Gibson and Earley, 2007). This is primarily due to the fact that team members are more likely to engage in learning behaviors when they share a belief that the team can do anything it sets out to accomplish (Edmondson, 1999). As a result, teams that rate high on efficacy are prone to persist in the face of a challenging goal, and even tend to push themselves to surpass such a goal when they come close to achieving it (Gully et al., 2002).

Research has shown how leadership can enable team efficacy. For instance, one way is to embody the belief that the team is capable of achieving good performance—especially shortly after team formation, since teams have little information to support such assessments (Pescosolido, 2001). Likewise, designing the team’s work in order to achieve early wins is another way for leaders to facilitate the emergence of team efficacy (Lester et al., 2002). Finally, leaders can closely monitor goal achievement to counter the negative effects associated with high levels of team efficacy (Rapp et al., 2014).

Efficacy can be said to be rooted in individuals’ beliefs (Bandura, 1997). Like goal orientation, such beliefs aggregate and can be focused on teams, and team scholars have picked up on this assumption (Gully et al., 2002). Also like goal orientation, they can represent proclivities and habits. They too are likely to have inertia, and to have a persistent influence on individual and team activities even when such beliefs are not actively being discussed. Efficacy thus fits the profile of a construct with inertia.

### Interplay Among Team Emergent States

Each of these four emergent states contributes significantly to team learning. However, they have usually been studied in isolation from each other. There are two reasons to be concerned about this. The first is that when it comes to the levels of team states, it is not always “more is better.” For example, high levels of cohesion have detrimental effects due to increased pressure for conformity (Lott and Lott, 1965; Hackman, 1976). Alternately, if team members believe *too* strongly in their ability to accomplish a task (efficacy), theory suggests that they can succumb to overconfidence and complacency (Gist, 1987). They tend to make poorer decisions by taking uncalculated risks, spending less time on information-processing activities, and rejecting negative feedback (Whyte, 1998). While such curvilinear relationships have not been investigated with respect to psychological safety, it would not be hard to imagine a team where effectiveness suffers because mistakes are so welcome. Similarly, there are contexts where performance orientation is more appropriate (Alexander and Van Knippenberg, 2014). The bottom line is that each emergent state has an optimum setting that may change with task and context.

This leads to the second point, efforts to influence one state may affect the utility of the others. For example, moderate level of team efficacy is recommended for teams to engage in learning behaviors that enhance performance (e.g., Tasa and Whyte, 2005), unless they monitor their goal closely—then, high level of team efficacy is beneficial (Rapp et al., 2014). Such contingencies mean that beneficial effects of one state might be counteracted by negative effects on another. It would explain why some researchers find a positive relationship between cohesion and team learning (e.g., Schippers et al., 2008), and others find no relationship (e.g., van Ginkel and van Knippenberg, 2008). What is unknown is whether some attempts to increase cohesion might not cancel out the benefits by also increasing a negative effects like groupthink (Janis, 1972). The bottom line is that if team emergent states affect each other, then research needs to address how to manage an equilibrium among them in order to maximize positive behaviors and outcomes such as learning in teams. We lack such an understanding of how the four team emergent states *collectively* influence team learning.

Research on the dynamics of teams is still in its infancy (Bowers et al., 2017), and conceptual work must therefore take a step forward and develop more dynamic models of team learning (Bell et al., 2012). Inertia is a foundation for dynamics – without inertia there is no way for the past to influence the future (Cronin and Vancouver, 2018). Above we have discussed why each emergent state could exhibit inertia. Yet to truly understand the *dynamics* of TLC, we must consider the feedback loops within the system. That is, how the change to emergent states produced by some leadership action may set into motion a causal chain that loops back to perpetuate or even reinforce the current conditions. Such feedback loops can diminish the intended effect of leaders’ actions or even worsen the problem via unintended consequences. What leaders really need to do is to promote virtuous cycles within the system. In all cases, one cannot control a system by focusing only on one part of it (i.e., one emergent state).

To be clear, when we discuss feedback, we are talking about circular chains of causality (Cronin and Vancouver, 2018). Feedback loops are what Marks et al. (2001) and others (e.g., Ilgen et al., 2005) have recognized as inherent in teams: An “output” at time 1 becomes the “input” at time 2. Such feedback is how non-linear growth and change can continue within a system even after a leadership action (or any other process intended to affect the team) has stopped. Feedback when coupled with inertia is also how systems as a whole resist change. To articulate how to control systems with inertia and feedback, it is often helpful to model them as *stock and flow systems* (Forrester, 1968). A stock is like a tank that maintains its water level over time unless it is filled or emptied. Thus it has inertia like other emergent states. But importantly, the stock and flow structure highlights that what causes TLC to increase may not be what causes it to decrease—the inflow to TLC can represent a different set of processes or actions from the outflow (Cronin and Vancouver, 2018).

This decoupling of inflows from outflows allows for greater prediction and control of TLC both within and between emergent states. For example, efficacy opens the inflow to TLC, for example, by increasing the motivation to perform. Yet after a certain point, efficacy might also open the outflow to TLC as well, albeit through a different process such as the discarding of new knowledge (i.e., “our way works, why would we change it?”). Such a characterization still fits with the conceptualization of efficacy, but it suggests that to control the system a leader should focus on counteracting the tendency to ignore new knowledge. The broader point is that the emergent states act as a collective to alter the inflow and outflow to TLC.

The systems view implies that to truly understand how leadership can manage TLC, research must conduct studies that will simultaneously monitor the equilibration among the different emergent states. To use another analogy, consider a vegetable garden. To achieve the highest yield, the gardener must balance soil quality, sunlight, watering, and pest control. The relative levels of all these factors in concert determine the garden’s potential to produce a healthy crop. Moreover, addressing one factor might influence another (e.g., using pesticides might impair soil quality). Further, the relationships are not linear: Some watering is needed, but not too much, and this also depends on the amount of sunshine. TLC is like the yield of the garden. It represents the team’s potential to learn effectively, based on the current levels of the important factors that support or inhibit team learning. In many ways, leaders must be capable gardeners.

**FIGURE 1:**
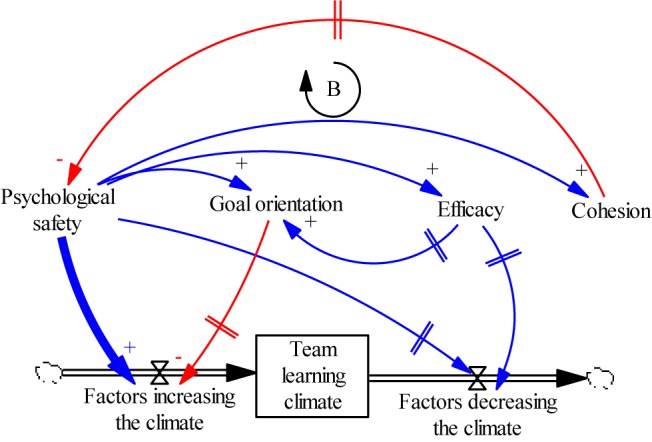
Dynamic model of team learning climate. Note that this model conforms to systems dynamics modeling conventions (Sterman, 2000). Boxed variables are stocks, and the hourglass shapes are flows. Cloud shapes represent factors exogenous to the model. Causal influence arrows are all directional, and denote either positive (blue) or negative (red) relationships. Arrows with “||” on the stem denote delayed influence (e.g., it may take time before goal orientation starts to influence the team learning inflow).

Figure 1 provides a more graphical illustration of the kinds of questions a systems view would warrant, and why these would be useful. The bottom of Figure 1 shows the stock of TLC with a single inflow and a single outflow, the arrows with hourglass symbols. Based on what we know about team learning, the inflow would represent experimentation and reflection processes (those that increase knowledge), while the outflow might represent forgetting and discounting processes (those that reject new knowledge). The emergent states are represented above the inflow and outflow arrows, and these have the capacity to influence each other as well as to open or close the flows. For simplicity, let us focus on psychological safety, and let us further assume that leaders are going to attempt to increase psychological safety through policy about the importance of always speaking up. The direct effect (represented by the bold arrow to the TLC inflow) should increase the rate of speaking up, which will encourage others in the team to do so as well, thus increasing the stock of TLC. Such an immediate effect can be tested and verified, but if one ignores the longer term effects, the understanding of the utility of this policy is incomplete.

For one thing, thinking about the growth of TLC over time leads one to realize that it would not be reasonable to expect that psychological safety will increase TLC forever. There is likely to be some control function, possibly emanating from the limits on psychological safety itself, that could eventually cause diminishing returns on the accumulation of TLC. We might conjecture that people will get used to the policy of speaking up, and thus its influence on behavior will fade over time as it becomes taken for granted. Alternately, after a certain point, psychological safety may start to decrease TLC if teammates feel no need to consider their ideas before voicing them; it may lead to a kind of information overload. This kind of influence is represented by the arrow from psychological safety to the outflow of TLC, and may only emerge after psychological safety grows to a certain point, which is why there is a delay mark (||) on the arrow.

As we discussed above, psychological safety can affect or be affected by the other states as well (Harvey et al., in press). These would be represented by the other curved arrow in Figure 1. Perhaps more important is that such effects can be delayed and can have second and even third order effects on TLC (i.e., the effect of psychological safety on TLC goes through two or three pathways). Consider first that while psychological safety can increase cohesion, as cohesion grows beyond a certain point it may increase conformity pressures which loopback to limit psychological safety (Kahn, 1990; Edmondson, 2018). This is a balancing loop (denoted by B). This would be another way that the impact of the policy that encourages speaking up might fade over time (as cohesion grows).

Sometimes the second order effects are harder to identify. Psychological safety may lead to increased efficacy, and as we discussed above, this might lead to overconfidence that decreases TLC as team members reject new knowledge (Rapp et al., 2014). This effect might also be delayed (represented by the two perpendicular lines on the arrow) because efficacy takes time to grow. However, once the effect of overconfidence surfaces and TLC starts to decrease, it may cause leaders to try to further increase psychological safety. Yet this will not fix the problem, and because of the delay between the change to psychological safety and the effect of overconfidence, leaders might overlook efficacy as the cause of the problem.

As the feedback loops get longer and causes and effects become more distal in time, the potential for perverse outcomes increases. Continuing with our example, if psychological safety improves efficacy, it might eventually change the goal orientation to a performance one (especially if performance is rewarded and the team gets used to “winning”). This is a second order effect that might produce the third order effect whereby performance orientation reduces the willingness to experiment and possibly fail, thus shutting the TLC inflow.

The important point about a systems view is that all of these things may co-occur. Thus, while initially psychological safety is a boon to TLC, over time its influence becomes more limited because of increased cohesion, and possibly even detrimental if the dark side of efficacy and goal orientation takes over. Managing this system thus requires managing all four emergent states, not just one.

## Team State Monitoring

We posit that TLC is produced and maintained by the joint effects of psychological safety, learning orientation, cohesion, and efficacy; they collectively affect team members’ engagement in learning behaviors. Team leaders have been shown to influence each of these emergent states (e.g., Edmondson and Harvey, 2017), but the emergent states operate as part of a system. In Figure 1 we described how leadership actions targeted at any one emergent state can have multiple, and sometimes unintended, consequences. To further illustrate this interplay and the collective influence of the emergent states that bring about TLC, we use a vignette of a teamwork situation where a leader attends to team needs, influencing subsets of TLC and, as a result, team learning.

We use the vignette to draw from the systems view in relation to TLC in order to extend the leadership function of team monitoring to *team state monitoring*. Team state monitoring brings the essential lessons of the systems view (i.e., inertia, feedback loops, etc.) together in an operational theory of TLC. This is useful because team research has been almost silent about the monitoring of a set of emergent states as an equilibrium that needs balance, and the various ways in which leaders can influence such equilibrium. Even though team monitoring has been shown to have a positive effect on some emergent states when taken separately (LePine et al., 2008), the original focus of these studies has not been the monitoring of emergent states *per se*, let alone the dynamic interplay found in the equilibrium such as the one that TLC represents. As argued above and further illustrated in the vignette that follows, a leader’s action intended to enhance one emergent state may also influence the trajectory of several others.

As presented above, team learning is conceptualized as the behaviors team members adopt internally such as experimenting and reflecting, which help the team transform inputs such as new team members or a novel task environment into performance outputs. This cycle creates dynamics that can affect TLC. The vignette in Box 1 illustrates what leaders should consider if they are to be capable gardeners, cultivating team learning.

**Box 1. The challenge of team state monitoring.**

This vignette concerns a team of five nurses with a reputation for taking on new challenges to improve quality of care. One winter, the hospital faces an influx of new patients, and the team is asked to integrate two young newcomers to deal with it. During a team meeting, the two new nurses appear nervous as the rest of the team skim through the workload, and make adjustments to implement a new procedure. As the team disperses, its manager overhears senior members sharing doubts regarding the team’s ability to deal with the increased demands, since the new recruits are so inexperienced. Over the next few days, several problems crop up. Team members seem to lack the drive to deal with the heavy workload. The manager, noticing the drop in performance, decides to join the next team meeting in the hope of instilling some self-belief.

In the meeting, the manager quickly realizes that the team is experimenting with a new procedure. Thinking that such a change may be too challenging for the new recruits, she takes over. She underlines the exceptional workload the team is facing and the importance of showing full competence during such peaks. She highlights the monetary incentives management offer for good performance, and lists the strengths that should help the team succeed. A team member interjects to list the benefits of the new procedure, but the manager dismisses her point. She reiterates the experience and knowledge of the team, maintaining that it has everything it needs to deliver right away. Her words seem to energize the team members as they prepare for their next shift. The team channels its energy toward getting the job done, and proves equal to the surge in patients. Over the next few weeks, team members continue to pay close attention to the performance indicators, and start receiving accolades. The atmosphere within the team is changing, as nobody wants to report a mistake that would affect team performance. Some members start “forgetting” to report certain errors. Months later, management trials a digital technology aimed at improving global health by syncing information across organizations. Due to its exemplary performance, the team is chosen for the “pilot.” The manager invites the team to use the technology even if it makes things difficult at first, emphasizing the benefits for patients. The team members nod in agreement. On the ward, however, none of them is particularly excited about experimenting with the new technology, and they avoid it whenever they can. If they made mistakes, it would affect team performance—and nobody wants that. Unsurprisingly, the manager learns little from the pilot. Thus, she ends the next meeting by urging the team to give her feedback so she can adjust things before rolling out the technology. Yet, very little changes the following week...

This vignette shows a team with a strong learning orientation that struggles to integrate newcomers while dealing with a particularly demanding workload, and therefore starts doubting its capability to improve. The leader intervenes to enhance the team’s shared belief of efficacy, but in doing so she also impacts the goal orientation of the team (performance starts overriding learning) and psychological safety (team members are now afraid to speak up or report mistakes that would affect short-term performance). While the team can handle the additional workload, the increase in efficacy is ultimately detrimental to TLC. The team may be less prepared to adopt new routines than it was before the leader’s intervention, meaning that they fail to learn continuously and improve the quality of care at the hospital. Worse, if performance suffers, newcomers may be blamed (e.g., “We were innovators until *they* showed up!”).

**FIGURE 2:**
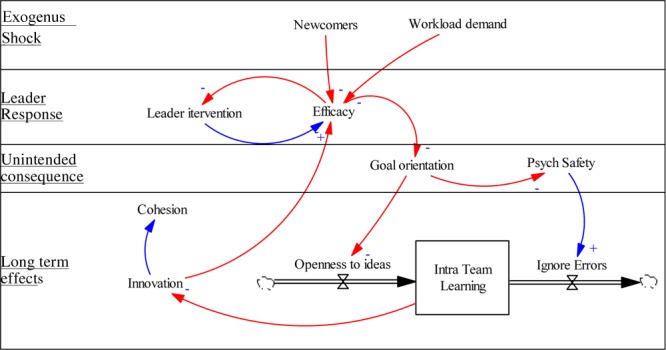
Dynamic model of team learning climate in the hospital vignette.

Using the systems view, we can model how this particular system might evolve in unexpected ways should the leader not monitor the four emergent states simultaneously. We display this in Figure 2. The exogenous shock of the higher workload and new team members reduces efficacy, and the leader responds to correct this. She continues to bolster efficacy (the positive link), and as it duly increases, she can scale back her intervention (the negative link). This is a type of self-efficacy control system, except the leader is the driver, rather than performance (cf. Vancouver et al., 2002). When the leader focuses on efficacy, it is easy to overlook the unintended effects on goal orientation and psychological safety. The variation in goal orientation increases resistance to change, decreasing the inflow to TLC. The decrease in psychological safety causes people to ignore errors from which they might learn, increasing the outflow from TLC. The joint effect is that TLC declines, making the team less innovative. Should this continue long enough, the decreased innovation may be blamed on the leader or even the newcomers, decreasing cohesion while also decreasing efficacy and perpetuating the cycle as the leader attempts to re-establish efficacy.

We recognize that newcomers do not always decrease efficacy, or alter goal orientation. The point is to illustrate how such a system works in this particular context, and to emphasize that if research is to discover the common patterns in TLC systems, research on TLC will need to start trying to model the systems, not just specific pieces of it. This is not merely a theoretical issue; it is a practical one as well. Understanding how emergent states can interact, balance and evolve gives leaders more flexibility in how they aim to sustain TLC. In the following section, we build on this viewpoint to develop avenues for future research and consider practical insights for leaders.

## Discussion

Taking a systems view on TLC opens up avenues for future research while also offering practical insights. Specifically, our work offers three main contributions to theory. First, we still know little about whether some of the emergent states that bring about TLC are more amenable to leadership interventions. Scholars have distinguished between task- and person-focused leadership (Koeslag-Kreunen et al., 2018) but TLC, while being rooted in persons’ beliefs, also relates to features of the team task. Thus it is unclear what focus would be recommended to influence TLC. One direction for future research is to examine whether the four emergent states are more (or less) likely to evolve over time—and, if so, under what conditions. Doing so may require a move away from cross-sectional designs toward special research designs and new measurement tools. For instance, experience sampling methodology (ESM), which demands that research participants complete several surveys over a relatively short period of time, could enable the investigation of the dynamics and coevolution we aim to delineate in this paper. Such *in situ* momentary assessments of team emergent states could show which ones are more or less event-contingent (see Kozlowski, 2015). The knowledge generated with this research can provide leaders with actionable insights into how to approach TLC monitoring.

Second, our work also provides grounds to think more deeply about who is best positioned to monitor TLC. The functional theory of leadership is inclusive when it comes to who should undertake leadership functions (Morgeson et al., 2010). Anyone inside and around the team can exert leadership, whether they assume a formal or informal role. Is there a difference between a longstanding team leader and a newly integrated team member intervening to influence TLC? This raises questions such as whether team members are more effective at monitoring emergent states, given their proximity to fellow members, or whether appointed leaders may provide greater stimulus to TLC trajectory by dint of their formal authority. Recent work by Koeslag-Kreunen et al. (2018) has shown that leadership from both formally appointed leaders and team members can influence team learning. Future research could look into team member interactions and how they might boost, maintain, or impair TLC. Computational methods would be particularly useful in leading such endeavors by modeling various team member characteristics and behaviors (see Cronin and Vancouver, 2018). The use of wearable wireless sensors designed to measure human social interactions is yet another way to give us cues about the respective influence of distinctive sources of leadership actions in real time (Kozlowski and Chao, 2018; Zhang et al., 2018). This could also shed light on the conditions underlying the changes—for example, whether team-level features such as task interdependence, or features associated with the work environment such as virtual communication, interact with monitoring practices to affect TLC.

Finally, while much has been written about TLC, what it actually represents has remained unclear. We hope to have provided more clarity to this important construct. However, we based our work on Bell et al. (2012) and therefore focused on psychological safety, goal orientation, cohesion, and efficacy. Other emergent states may need to be included in TLC. One avenue for future research in that direction is to validate TLC as a second-order construct, similar to what Mathieu et al. (in press) have done with the action, transition, and interpersonal processes of teamwork proposed by Marks et al. (2001). Researchers need to map the many emergent states that have proliferated throughout the past decades or so, put them under larger umbrellas (second-order constructs), and test them empirically. This likely means reducing the number of items used to measure each emergent state and reassessing validity (Smith et al., 2000), but this is necessary to start exploring the dynamics between these key constructs. Only then will team research be able to fully embrace the systems view that we propose here.

In terms of practical implications, taking a systems view on TLC can help managers interpret the potential multivariate effect of their actions. For instance, a manager who wishes to cultivate psychological safety by modeling openness and asking feedback from team members can affect the goal orientation, efficacy, and cohesion of the team depending on the content of the feedback that is provided and the exchanges that ensue. Training managers in systems thinking could be useful to develop their holistic conception of management practice and leadership, which goes beyond the logical thinking that is usually taught in business schools. In general, this should lead managers to better appreciate the complexity of their impact and reduce the impression of direct connectedness between their actions and the desired outcomes.

Thinking of TLC as an equilibrium that needs balance also brings the notion of time to the fore. It moves away from the perception of TLC as a starting point or a definite state represented as an intrinsic dialectical quality (learning vs. non-learning climate). Managers can then better understand why TLC is never a *fait accompli* and rather an enduring accomplishment that revolves around managing several emergent states over time. Going back to Senge (1990), this is at the foundation of the reflexivity and inquiry skills necessary for organizations to thrive over the long haul.

## Conclusion

Team scholarship has primarily focused on emergent states in isolation, limiting our understanding of the proper “milieu” among them or our insights into how they operate jointly. Therefore, it is not immediately apparent how the various emergent states differ from each other, or where they overlap (Bell et al., 2012). This has led to scholarship that does not always take into account the complexity of the bundle of emergent states present in TLC. We hope that our efforts in this paper offers the opportunity for scholars to take more of a systems view in their research on TLC, and for leaders to embrace the complex, yet crucial, role they play in continuously shaping team members’ beliefs. This is all very challenging, but the rewards are well worth it, as teams continue to flourish in science and in the field.

## Data Availability

No datasets were generated or analyzed for this study.

## Author Contributions

JFH and MC developed the research idea and wrote most of the manuscript. PML assisted them on parts of the manuscript, particularly the literature review.

## Conflict of Interest Statement

The authors declare that the research was conducted in the absence of any commercial or financial relationships that could be construed as a potential conflict of interest.
